# Microanalytical Characterization of an Innovative Modern Mural Painting Technique by SEM-EDS, NMR and Micro-ATR-FTIR among Others

**DOI:** 10.3390/molecules28020564

**Published:** 2023-01-05

**Authors:** Pablo Aguilar-Rodríguez, Sandra Zetina, Adrián Mejía-González, Nuria Esturau-Escofet

**Affiliations:** 1 Instituto de Química, Universidad Nacional Autónoma de México, Mexico City 04510, Mexico; 2 Instituto de Investigaciones Estéticas, Universidad Nacional Autónoma de México, Mexico City 04510, Mexico

**Keywords:** pMMA, multianalytical characterization, painting medium, mural painting art, NMR, micro-ATR-FTIR

## Abstract

During the 20th century, modern painters experimented with different mediums and painting techniques, one of them was Rafael Coronel in his mural painting, *Paisaje Abstracto* (*Abstract landscape*). The painting was created with a peculiar pouring technique and an unknown binding medium; ageing produced fractures and severe conservation problems. Therefore, the characterization of the painting medium became an urgent matter in order to understand the current condition of the painting and to develop a proper treatment. The aim of this research was to characterize the chemical composition and painting technique of *Paisaje Abstracto*. To approach this goal two microsamples were taken and analyzed by optical microscopy (OM), scanning electron microscopy (SEM) with energy dispersive spectroscopy (EDS), nuclear magnetic resonance (NMR) spectroscopy, attenuated total reflection Fourier transform infrared spectroscopy (ATR-FTIR), micro attenuated total reflection Fourier transform infrared spectroscopy (micro-ATR-FTIR) and gas chromatography/mass spectrometry (GC/MS). The analysis allowed for the identification of cadmium sulfide (CdS) and titanium dioxide (TiO_2_) as inorganic pigments; aluminosilicate fillers; poly(methyl methacrylate) (pMMA) as a binder; MMA monomer, red organic pigment PR181; benzoyl peroxide, dibutyl phthalate and 1-octadecanol as organic additives. This study presents an innovative painting technique with pMMA, a medium not commonly used by artists, which was probably polymerized onto the painting support.

## 1. Introduction

*Paisaje Abstracto* (*Abstract landscape*) is a transportable mural painted in 1964 by Rafael Coronel (1932–2019) to decorate the hall that leads to the Library at the Museo Nacional de Antropología (MNA, National Museum of Anthropology), in Mexico City. Rafael Coronel created an abstract composition that presents large areas of bright colors emerging from darker regions; a horizontal red haze at the center dominates the rectangular format. The painting technique is unusual, the composition lies over the texture qualities but there are no visible brushstrokes, as if the drying of the binding medium was slow. The resulting rough surface recalls a landscape, a coarse bubble-like crust with elevations and valleys. It seems to be created by pouring red, yellow and blue dense painting layers intermingled with black, gray and dark ocher thin layers, in a manner that recalls the experiments with automatist painting of American abstract expressionists or French tachistes.

The painting is not signed, and no documentation has been found at the museum archives about the author or the materials used by the artist. Between 1963 and 1964, at an early stage in his career, Rafael Coronel was experimenting with abstraction, but also with figurative painting, and it is evident that he was searching for new materials and techniques. Some sketches, painted with synthetic binding medium over canvas, were found at the artist collection; they bear the title Abstracción (Abstraction) and present a red irregular area over a black rough surface. At the same time that the *Paisaje Abstracto* was painted, Coronel produced another well documented figurative painting, El mundo espiritual de los mayas peninsulares (The Spiritual World of Peninsular Mayas), for the same museum with a rather traditional brushwork technique using acrylic painting.

These paintings are part of the ambitious project developed by Architect Pedro Ramírez Vázquez for the creation of the acclaimed MNA in 1964. Developed under Adolfo López Mateos’ government (1958–1964), it became a symbol of Mexico’s cultural heritage [1]. The MNA project combined contemporary monumental architecture, innovative displays and Modern art, with the intention of giving a prominent place to Mesoamerican and indigenous art, but also to present Mexico as a cosmopolitan country through the inclusion of Modern tendencies [1].

The MNA Modern art project included sculptures, murals and assemblages, in which 23 artists associated with diverse and even contrasting artistic tendencies participated: social realists, advocates of pure painting or abstraction, and even surrealists, in an array of the proposals in conflict between the 1950s and 1960s in Mexico. Rafael Coronel pertained to the younger generation, sometimes called Ruptura, a term that inefficiently defines several divergent artistic groups and artists that rebelled against the Mexican School of Painting or muralism and their social realism [2]. Many of the younger painters, such as Rafael Coronel, preferred to be understood as individuals rather than as a group. Coronel was sometimes affiliated with the proposals of the New Presence or interiorista manifestos, an introspective current that evocated personal emotions through the violent application of painting, but was neither associated with abstraction or figuration [3].

The younger generation of Mexican painters rejected social realism associated with muralism, but were interested in David Alfaro Siqueiros’s innovations with industrial binding mediums and techniques such as cellulose nitrate lacquers and spray guns, commonly used for the automotive industry. They appreciated the diversity of possibilities that offer synthetic mediums to create textures, volumes and color contrast, and in that sense, *Paisaje Abstracto* seems to be an experiment with industrial binding mediums to produce an abstract composition.

In this paper, we characterize the composition and painting technique of *Paisaje Abstracto*, which was painted with an unknown synthetic medium over a wood panel support; the painting layers are rigid and detached due to the expansion of the wood. During the last conservation process, two samples were provided by restorers in order to understand the materials and techniques used by the artist with the intention of designing the treatments. This paper presents the study of the binder; the additives, aggregates, and organic and inorganic pigments of these two samples that were analyzed by Optical Microscopy (OM), Scanning Electron Microscopy–Energy Dispersive X-ray Spectroscopy (SEM-EDS), Nuclear Magnetic Resonance (NMR) Spectroscopy, Attenuated Total Reflection Fourier Transform Infrared spectroscopy (ATR-FTIR), Micro Attenuated Total Reflection Fourier Transform Infrared spectroscopy (micro-ATR-FTIR) and Gas Chromatography/Mass Spectrometry (GC/MS).

The study is also aimed at examining the functionality, performance and time saving of scientific instruments suitable for the analysis of works of art of the period, many made with synthetic or innovative unknown materials. The selected methodology could be proposed as a protocol to be used for the art historical study and conservation of modern paintings created during the second half of the twentieth century.

Regarding the NMR spectroscopy technique, is important to mention that it is a powerful and frequently used technique in many fields, from basic to applied sciences, to characterize both molecular and supramolecular structures of organic and inorganic compounds. The main disadvantage of NMR spectroscopy is its low sensitivity compared to chromatographic separation coupled with any kind of mass spectrometry detection. This is an essential issue for cultural heritage analysis due to the limited amounts of samples available. However, the sensitivity of NMR spectroscopy has increased enormously because of recent improvements in hardware and instrumental advances. Therefore, in recent years, NMR spectroscopy as a tool for characterizing complex mixtures has increased, particularly in the field of cultural heritage, as it provides valuable insights in the identification of organic materials such as waxes, paint binders, pigments, additives and organic degradation products [4,5,6,7,8,9].

## 2. Results

### 2.1. Description of the Technique and Observations of the Conservation State

**Figure 1:**
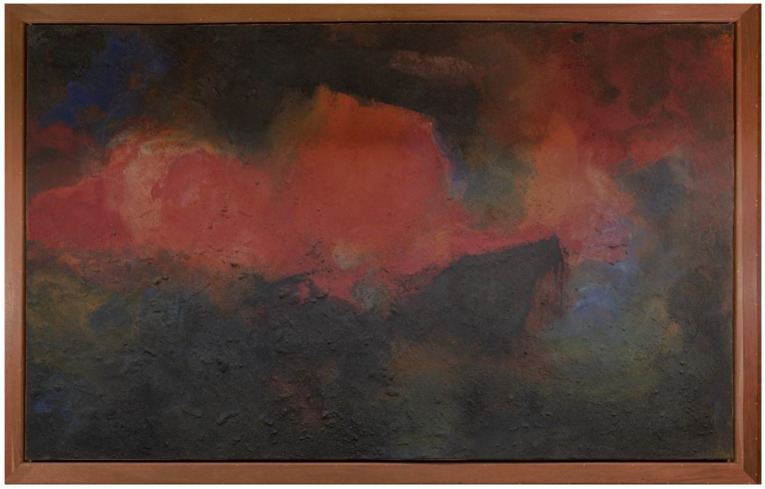
Photo of *Paisaje Abstracto* by Rafael Coronel after intervention. Photo: D.R. ©Digital Archive of the Collections of the National Museum of Anthropology, INAH, Canon, 2018.

The painting structure of *Paisaje Abstracto* (Figure 1) is quite simple; the medium and pigments were applied directly onto a hardwood plywood panel, formed by two plywood sheets that were sustained by a reticular wood stretcher. Originally, a wooden frame reinforced the structure. These kinds of hardwood panels, of monumental dimensions (3.90 × 2.40 m) were produced by the museography staff and are similar in structure and dimensions to other paintings in the museum [10].

The painting layer was possibly applied over the support placed in a horizontal position. It seems more like the materials were poured over the surface, creating textures and considerable elevations, a kind of topographic relief formed by the unevenness of the surface. When samples were observed under the magnifying lenses, the surface had a bubble texture of spheres. Under UV lighting, the surface of the painting presents strong fluorescence in red, dark brown and yellow areas [10]. The palette is restricted: blue, yellow, orange, red, dark ochre, black and white, were applied by areas. The intense colors emerge from a darker foreground; multiple textures increase the formation of a layered, volcano-like structure. The painting layer has an average of 1 cm of thickness with elevations that may protrude as high as 3 cm.

Only one side of the original frame was preserved; the stretcher was in good condition, but the plywood panels had deformations that detached two sections of the plywood from the stretcher and formed a concavity. The upper section to the right of the painting was separated by about 1.30 m and was detached almost 6 cm from the stretcher [10].

Possibly the loss of the frame, the changes in relative humidity and the particularly rigid, uneven and thick painting layer, produced a severe deformation of the plywood panels, and consequently a bulge detachment and the fracture of the painting layers in the heavier areas. The fractured and split areas from the support were veiled by conservators with non-woven fabric to prevent them from falling [10].

The rigidness of the painting layer and its lack of flexibility compromise its adhesion to the wood panel support. On the other hand, the wood support is reactive to the conditions of relative humidity and temperature, which alter its dimensions cyclically. The chemical composition of the painting layer forms a rigid solid that will not meet the dimensional changes of the wood panel.

### 2.2. Optical Microscopy Study

**Figure 2:**
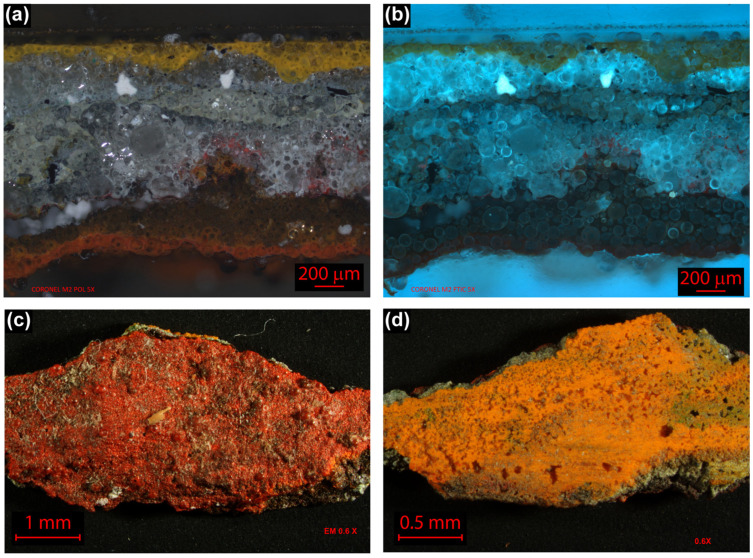
Cross section micrograph of the mural *Paisaje Abstracto* sample (area studied approximately 2100 × 1400 microns, magnification: 5×). (**a**) Polarized light; (**b**) UV filter, 460 to 490 nm (FITC); (**c**) sample without preparation, front surface area (approximately 3 × 6 mm); (**d**) back of the sample. Images (**a**,**b**), the cross section micrographs present the paint layers in reverse order (those closest to the panel are at the top).

Figure 2a is a micrograph of the cross section of the sample with polarized light, where six layers can be distinguished: the first layer is yellow, followed by a greyish layer, then a dark red color and finally the red surface paint layer. In all the samples, an agglomeration of spherical structures is observed. Figure 2b shows the cross section of the sample with a UV filter from 460 to 490 nm, where the intermediate gray layer, identified in Figure 2a, presents different fluorescence caused by the overlay of rich binder layers and fillers, and pigments layers. Whitish regions with increased fluorescence that could be attributed to pigments or inorganic fillers are evident. The artist did not use a ground preparation for this mural; he applied, directly onto the support, the different layers of paint, where the surface paint layer was red (obverse side) (Figure 2c) and the first was a yellow layer (reverse side) (Figure 2d). The yellow painting layer has a flat surface because this side was attached to the panel.

### 2.3. Elemental Composition Analysis by SEM-EDS

**Figure 3:**
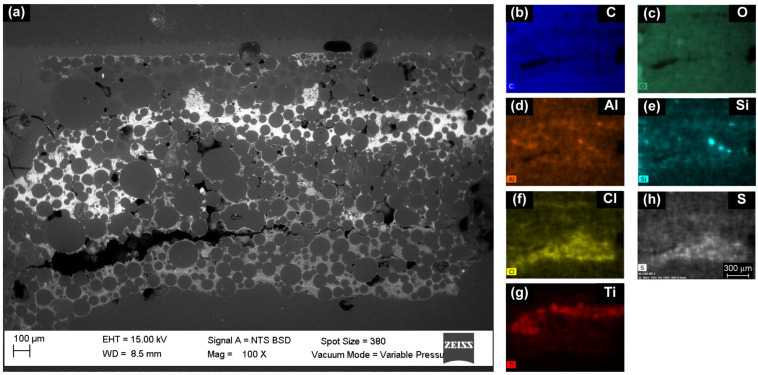
(**a**) SEM micrograph (BSE, 15.0 kV, 100×, area studied approximately 3000 × 1800 microns). Elemental mappings (EDS, 15.0 kV, 100×) of predominant elements: (**b**) C, (**c**) O, (**d**) Al, (**e**) Si, (**f**) Ti, (**g**) Cl and (**h**) S.

Figure 3a shows the backscattered electrons (BSE) micrograph of the *Paisaje Abstracto* cross-section, in which low-contrast circular regions with a size between 20 and 150 m predominate, which are embedded in a heterogeneous matrix.

The matrix is composed of an organic material due to its high presence of C and O (Figure 3b,c). The presence of inorganic components with different geometries is observed. The yellow pigment was identified as cadmium yellow due to the presence of Cd and S in the same region (Figure S1). The presence of Al and Si (Figure 3d,e) in the same regions suggest the presence of an aluminosilicate filler. Ti (Figure 3f) was related to white titanium pigment TiO_2_. In the red surface region of the sample, shown in Figure 2a, no elements that could be related to an inorganic pigment were detected. The predominant presences of Cl and S (Figure 3g,h) suggest the presence of an organic pigment.

### 2.4. Organic Compound Elucidation by NMR

The structure of the major organic compounds present in the sample of *Paisaje Abstracto* were elucidated by the interpretation of one-dimensional (^1^H and ^13^C) and two-dimensional (COSY, ed-HSQC and HMBC) NMR spectra. COSY spectra allowed us to identify homonuclear correlations between vicinal hydrogens separated by two or three bonds. The HSQC was used to determine correlations between directly bonded hydrogen to carbon atoms. Additionally, thanks to the multiplicity-edited HSQC, it was possible to differentiate between CH/CH_3_ and CH_2_ groups. The HMBC gives heteronuclear correlations between hydrogen and carbon atoms separated by two, three and, sometimes in conjugated systems, four bonds, giving connectivity information.

The spectra of ^1^H, ^13^C, ed-HSQC, HMBC and COSY are reported in Figures S2–S12. Based on the analysis of the NMR spectra, the compounds in the following subsections were elucidated (see Table 1 for NMR data and molecule structure). Atom numbering is indicated in each structure only for assignment purposes. The analytic process to reach them is presented below.

#### 2.4.1. Binding Medium

The principal component in the sample is the binder which was identified as pMMA due to the HMBC correlation from the methylene protons broad signal H-2 ($\delta_{1_{H}}$ 1.81 ppm) with three methyl carbon signals C-1 ($\delta_{13_{C}}$ 16.66, 18.82 and 21.24 ppm), a methylene carbon C-2 ($\delta_{13_{C}}$ 51.24–54.48 ppm), a quaternary carbon C-3 ($\delta_{13_{C}}$ 44.74 ppm) and a carbon in a carbonyl group C-4 ($\delta_{13_{C}}$ 176.26–178.44 ppm) (Figure S4). The carbonyl group was identified as an ester due to the HMBC correlation from the methyl protons signal H-5 ($\delta_{1_{H}}$ 3.60 ppm) to C-4. All this indicates that the binder is exclusively composed of atactic pMMA due to the tacticity observed [11].

#### 2.4.2. Monomer

The MMA monomer was also identified due to the HMBC correlations from methyl protons H-4 ($\delta_{1_{H}}$ 1.94 ppm) to a carbonyl C-2 ($\delta_{13_{C}}$ 167.86 ppm), a quaternary carbon C-3 ($\delta_{13_{C}}$ 137.38 ppm) and a methylene carbon signal C-5 ($\delta_{13_{C}}$ 125.61 ppm), which indicate the presence of a methacrylate (Figure S5). The HMBC correlation, from methyl protons H-1 ($\delta_{1_{H}}$ 3.75 ppm) to C-2, confirmed the identification of the MMA monomer.

#### 2.4.3. Catalyst

The catalyst benzoyl peroxide was elucidated from the COSY correlation between the aromatic protons H-1 ($\delta_{1_{H}}$ 7.60 ppm) and H-2 ($\delta_{1_{H}}$ 7.47 ppm) and between H-2 and H-3 ($\delta_{1_{H}}$ 8.08 ppm) (Figure S6). The HMBC correlations from H-3 to aromatic carbons C-1 ($\delta_{13_{C}}$ 133.69 ppm) and C-3 ($\delta_{13_{C}}$ 130.28 ppm) and a carbonyl signal C-5 ($\delta_{13_{C}}$ 167.79 ppm) confirmed the presence of benzoyl peroxide which is commonly used as a catalyst (Figure S7).

#### 2.4.4. Plasticizer

The HMBC correlation from the aromatic protons H-2 to aromatic carbons C-1 ($\delta_{13_{C}}$ 131.03 ppm) and C-3 ( 132.63 ppm), and a carbonyl signal C-4 ($\delta_{13_{C}}$ 167.91 ppm), indicate the presence of a symmetric ortho disubstituted aromatic system (Figure S8). The aliphatic system of the molecule was identified with the COSY correlation between methylene protons H-5 ($\delta_{1_{H}}$ 4.31 ppm) and H-6 ($\delta_{1_{H}}$ 1.72 ppm), H-6 and H-7 ($\delta_{1_{H}}$ 1.44 ppm) and H-7 and methyl signal H-8 ($\delta_{1_{H}}$ 0.96 ppm) (Figure S9). The HMBC spectra confirmed the presence of dibutyl phthalate with the correlation from methylene protons H-5 to carbonyl signal C-4, and methylene carbons C-6 ($\delta_{13_{C}}$ 30.74 ppm) and C-7 ($\delta_{13_{C}}$ 19.28 ppm).

#### 2.4.5. Additive

A linear chain alcohol structure was elucidated from COSY correlation between methyl protons H-1 ($\delta_{1_{H}}$ 0.88 ppm) and methylene H-2 ($\delta_{1_{H}}$ 1.29 ppm), H-2 and methylene H-3 ($\delta_{1_{H}}$ 1.25 ppm), methylene H-5 ($\delta_{1_{H}}$ 1.34 ppm) and methylene H-6 ($\delta_{1_{H}}$ 1.29 ppm), and H-6 and methylene H-7 ($\delta_{1_{H}}$ 3.64 ppm) (Figure S10). The HMBC correlation from H-4 ($\delta_{1_{H}}$ 1.25 ppm) to methylene carbon C-2 ($\delta_{13_{C}}$ 22.80 ppm) and from H-5 and H-6 to methylene carbon C-4 ($\delta_{13_{C}}$ 29.40 ppm) shows the linear connectivity from C-1 to C-7 (Figure S11).

#### 2.4.6. Pigment

The HMBC correlation from methyl proton H-9 ($\delta_{1_{H}}$ 2.73 ppm) to aromatic carbons C-3 ($\delta_{13_{C}}$ 125.09 ppm), C-4 ($\delta_{13_{C}}$ 143.63 ppm) and C-5 ($\delta_{13_{C}}$ 129.05 ppm), with correlation from H-5 ($\delta_{1_{H}}$ 7.09 ppm) to C-3 and C-7 ($\delta_{13_{C}}$ 121.98 ppm) (Figure S12), indicate the presence of a tetra-substituted aromatic system which can be attributed to red pigment PR181 due to the identification of Cl and S in SEM-EDS (Figure 3g,h) in the red regions of the cross section (Figure 2a).

### 2.5. ATR-FTIR Study

**Figure 4:**
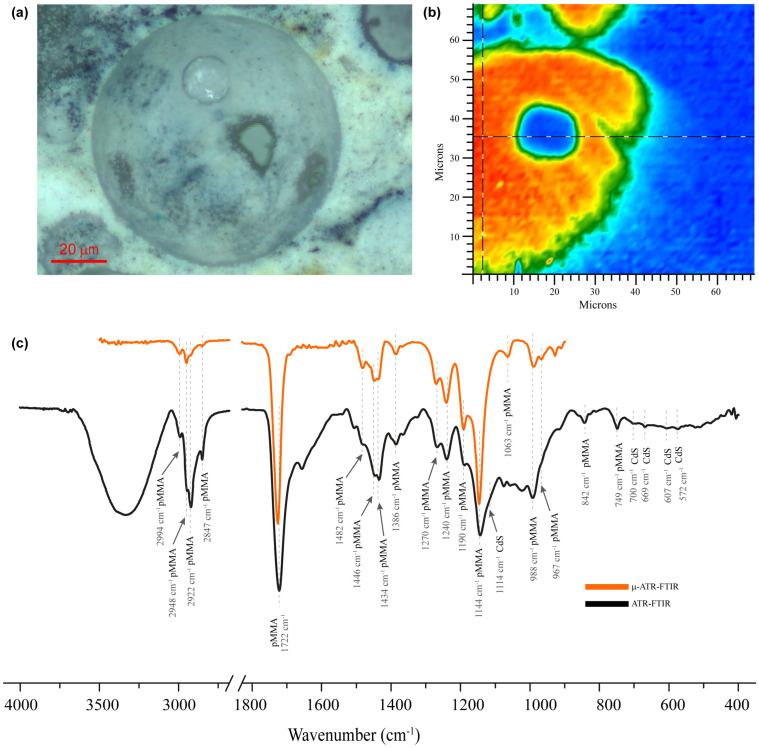
Cross sectional micrograph of the isolated layer yellow sample: (**a**) visible microscopic image, (**b**) chemical map displaying the intensity of the absorbance peaks at 2860 to 3040 cm^−1^, (**c**) comparison between ATR-FTIR (dark line) and micro-ATR-FTIR spectra obtained from the highlighted region in false color image (orange line).

ATR-FTIR analytical characterization was performed to identify the binder and pigments in the *Paisaje Abstracto* sample. The ATR-FTIR spectrum (Figure 4c) were assigned by relating them to the bands of the pMMA binder and these were compared with those of reference libraries. However, the bands of the characteristic groups of the additives, previously elucidated by NMR, were not observed due to their low concentration (see Table 2).

#### 2.5.1. pMMA

The presence of pMMA was confirmed by comparing the profile of the ATR-FTIR spectra and the absorbance of diagnostic peaks within the literature. The signals attributed to the binder were the carbonyl stretching mode at 1722 cm^−1^; the asymmetrical stretching mode of carbonyl group _a_(C-CO-O) at 1240 and 1270 cm^−1^; the asymmetrical stretching mode of ester group _a_(C-O-C-) at 1144 and 1190 cm^−1^; the C-H combination band of -CH_3_ at 2847 and 2922 cm^−1^; the band which included the symmetrical stretching mode _s_(C-H) of O-CH_3_ with _s_(C-H) of -CH_3_ and asymmetrical stretching mode _a_(CH_2_) at 2948 cm^−1^; _a_(C-H) of O-CH_3_ with _a_(C-H) of -CH_3_ at 2994 cm^−1^; the ones related to symmetrical bending modes _s_(C-H) of -CH_3_ and O-CH_3_ at 1386 and 1434 cm^−1^; the signal related to asymmetrical bending _a_(C-H) of -CH_3_ at 1446 cm^−1^; the methylene scissoring mode signal (CH_2_) at 1482 cm^−1^; the signals that correspond to rocking mode (C-H) of CH_2_, CH_3_ and O-CH_3_, respectively, at 842, 967, 988 cm^−1^; and the skeletal stretching mode bands (C-C) at 749, 1063 cm^−1^ [12,13].

#### 2.5.2. Organic Pigment

The ATR-FTIR spectra obtained from the red layer (Figure S13) shows bands associated with the red pigment PR181 [14,15]. Some bands that are present in the ATR-FTIR reference spectrum of the red pigment PR181 can also be associated with pMMA vibrations. Those bands are methyl group scissoring (H-C-H) at 1434 cm^−1^; combination band at the 1386 cm^−1^ band of methyl group scissoring (H-C-H), benzene ring bending (C-C-H) and symmetrical stretching _s_(C-C-C); combination band at 1240 cm^−1^ which includes the bending of benzene ring (C-C-H), asymmetrical stretching of thiophene ring _a_(C-C-C), symmetrical stretching of benzene ring _s_(C-C-C), and symmetrical bending of methyl groups _s_(H-C-H); combination band at 1190 cm^−1^ that includes the bending of benzene ring (C-C-H), asymmetrical stretching of thiophene ring _a_(C-C-C and C-C-S), symmetrical stretching of benzene ring _s_(C-C-C); and a band identified at 842 cm^−1^, of which its vibration was not assigned. However, and based on the compounds identified by NMR, the ATR-FTIR bands that can only be assigned to the red pigment and thus confirm its presence in the sample are band at 1654 cm^−1^ related to carbonyl in thiophene ring (C=O), stretching of benzene and thiophene ring (C-C-C) band at 1562 cm^−1^; combination band at 1294 cm^−1^ that includes the asymmetrical stretching of benzene ring _a_(C-C-C), symmetrical stretching of thiophene ring _s_(C-C-C) and symmetrical bending of methyl groups _s_(H-C-C-C-H); combination band at 1096 cm^−1^ scissoring of benzene ring (H-C-C-C-H), carbon-chloride stretching (C-Cl), symmetrical stretching of benzene ring _a_(C-C-C), benzene ring and methyl group rocking (H-C-C-C-H) and asymmetrical stretching of thiophene ring _s_(C-C-S), 1048 cm^−1^ thiophene ring stretching (C-C); combination band at 823 cm^−1^ of benzene ring rocking (H-C-C-C-H), symmetrical stretching of thiophene ring _s_(C-S-C) and carbon-chloride stretching (C-Cl); the combination band at 468 cm^−1^ of benzene ring and methyl group symmetrical bending _s_(H-C-C-C-H), thiophene ring bending (C-C-O) and carbon-chloride stretching (C-Cl); and a band at 781 cm^−1^, of which its vibration was not assigned.

#### 2.5.3. Inorganic Pigment

The presence of CdS pigment was confirmed with the bands related to the Cd-S bond at 700, 669 and 607 cm^−1^. Additionally, bands related to cadmium yellow PY37 ATR-FTIR spectra (1114 and 572 cm^−1^) were attributed to this pigment [16,17,18,19].

### 2.6. Micro-ATR-FTIR Analysis

In order to clarify the molecular composition of the spheres, a micro-ATR-FTIR mapping of the cross section of the sample was carried out. Figure 4a shows the optical microscope image of the mapped region and Figure 4b shows the chemical map obtained by integrating the bands at 2860 to 3040 cm^−1^. Figure 4c shows the micro-ATR-FTIR spectra of the cross section of the sample, indicated in Figure 4b, and the stacked ATR-FTIR spectra obtained from the sample surface. The chemical map in Figure 4b confirms that the spheres are mainly composed of pMMA, both on their surface and inside.

### 2.7. GC/MS Analysis

The molecular weights of the volatile and thermostable compounds were confirmed by GC/MS (see chromatogram in Figure S14 and mass spectra in Figure S15a–d). The compounds identified were Benzoyl peroxide (RT = 11.38 min), dibutyl phthalate (RT = 23.38 min), 1-octadecanol also known as Stearyl alcohol (RT = 24.77 min) and the red pigment PR181 (RT = 40.84 min).

## 3. Discussion

Commercial artistic paintings are typically copolymerized MMA monomers with ethyl or *n*-butyl acrylate and methacrylate [19]. The finding of pMMA homopolymer as the binder of *Paisaje Abstracto* was unanticipated. The use of pMMA as a material for artistic purposes is uncommon and has not been reported before in artistic paintings.

The lack of organic and inorganic additives suggests that Rafael Coronel manufactured his own paint for this mural, mixing methyl methacrylate, poly(methyl methacrylate), benzoyl peroxide, aluminosilicate fillers, additives and organic and inorganic pigments. The layer sequence of polymer and pigment observed under the microscope support this statement. It seems that the artist poured mixtures of different colors with the see chromatogram in Figure S14 and mass spectra in Figure S15a–d support placed horizontally, producing elevations in the surface.

Based on the organic compounds identified by NMR, it could be proposed that Coronel used an application process similar to the Kulzer patented method to produce dental prosthetics in the mid-1930s [20]. Moreover, the alcohols most used in coating formulation are those with a maximum length of 5–8 carbons as biocides [21]. Therefore, the identification of 1-octadecanol could be more related for surgical or dental prostheses and dental repairs, as it was patented by Bayer Ag in 1952 [22].

Coronel may have used a higher proportion of pMMA/MMA, which could explain the formation of pMMA spheres [23]. The lack of miscibility of the polymer in the monomer can inhibit the chain transfer reaction of the monomer radical to the prepolymerized pMMA which did not allow optimal curing [24]. A modification in the pMMA/MMA proportion could affect the mechanical properties of the painting, especially the compressive strength, yield stress and compressive modulus [25,26].

## 4. Material and Methods

### 4.1. Sampling

During restoration, due to the dimensional changes (by expansion or contraction) of the wood panel, two microsamples were detached from the upper right corner. Conservators from the MNA provided the samples. Both samples had a red color on the outer surface and both had the same sequence of six layers: (i) yellow directly over the support, (ii) green, (iii) gray, (iv,v) two translucent ones, and (vi) red on top. In general, the painting layer was very prominent and thick; the stratigraphy of the samples measured approximately 2500 microns, but there were regions in the painting that were thicker than this section. The samples were inspected with an optical fiber microscope (Keyence, Osaka, Japan), then one sample section was reserved for chemical studies of isolated layers: yellow and red; the other sample section was embedded in Claro Cit©, a cold mounting acrylic resin produced by Struers (Roper Technologies, Sarasota, FL, USA), for the stratigraphic analysis.

### 4.2. Optical Microscopy

Samples were studied with an Axio Imager Z2 optical microscope (Carl Zeiss, Oberkochen, Germany) equipped with a Xenon arc lamp for UV fluorescence with 430–465 nm and 465–500 nm filters and a HAL100 light source in reflected light mode. The inspection under the optical microscope was performed over the cross sections.

### 4.3. Scanning Electron Microscopy—Energy Dispersive X-ray Spectroscopy

The electron micrographs were acquired with an EVOMA25 SEM (Carl Zeiss, Oberkochen, Germany) primarily with a backscattered electron detector. An accelerating voltage was applied between 15.0–17.0 keV, due to the organic composition of the sample and its sensitivity to be damaged by X-rays. The chemical elemental analysis of the samples was performed with an Energy Dispersive Spectroscopy microprobe, 30 mm (Bruker, Bremen, Germany) and PB/ZAF analysis. Samples were studied as cross sections. Double-stick carbon tape was used to attach and render the samples conductive. The images were taken using variable pressure (80 Pa) under nitrogen flow to avoid electrostatic charges on the samples’ surfaces.

### 4.4. Nuclear Magnetic Resonance Spectroscopy

NMR spectra were acquired with a Bruker Avance III HD 700 spectrometer (Bruker, Billerica, MA, USA) operating at 16.4 T (700 and 175 MHz for ^1^H and ^13^C frequency, respectively) equipped with a 5 mm z-axis gradient TCI cryoprobe. The ^1^H-NMR and 2D-NMR experiments were acquired at 298 K with standard pulse sequences from the Bruker library and processed with MestReNova software (v. 14.0, Mestrelab Research SL, Santiago de Compostela, Spain). The 2D-NMR experiments acquired were ^1^H-^1^H correlation spectroscopy (COSY), ^1^H-^13^C edited heteronuclear single quantum correlation spectroscopy (edited-HSQC) and ^1^H-^13^C heteronuclear multiple-bond correlation spectroscopy (HMBC). The chemical shifts (δ) are reported in ppm relative to the solvent resonance as the internal standard (CDCl_3_: δ^1^H = 7.26 ppm).

### 4.5. Attenuated Total Reflection Fourier-Transform Infrared Spectroscopy

ATR-FTIR was performed on samples without preparation with the Cary 600 spectrophotometer (Agilent Technologies, Santan Clara, CA, USA). The range used by the IR spectra was 4000–400 cm^−1^. Spectra were acquired with 128 scans at 4 cm^−1^ resolution. All data were processed with Origin software. The signal identification was compared with the published literature.

### 4.6. Micro-Attenuated Total Reflection Fourier-Transform Infrared Spectroscopy

Micro-ATR-FTIR analyses were performed on cross sections with an Agilent Cary 620 FTIR microscope (Agilent Technologies, Santa Clara, CA, USA) with a 64 × 64 Focal Plane Array (FPA) detector coupled to an Agilent Cary 660 FTIR spectrometer. Data were collected at 1.1 µm per pixel resolution using a micro Germanium Attenuated Total Reflection crystal accessory and processed using Agilent Resolutions Pro software (Agilent Technologies, Santa Clara, CA, USA). The Field of View (FOV) was 70 × 70 µm. Spectra were acquired in the spectral range between 4000 to 900 cm^−1^, performing 128 scans at 4 cm^−1^ resolution.

### 4.7. Gas Chromatography/Mass Spectrometry

Mass spectra were acquired using an Agilent 7890B gas chromatograph (Agilent Technologies, Santa Clara, CA, USA) equipped with capillary column HP-5ms cross-linked (5%-phenyl)-methylpolysiloxane (30 m × 0.25 mm × 0.25 μm) and an Agilent 5977A mass spectrometer (Agilent Technologies, Santa Clara, CA, USA) operated in positive mode with electron impact ionization (70 eV). The injector was set at 280 °C and the interface at 310 °C. The initial temperature for the GC column was 40 °C, held for 1 min, increased at 8 °C min^−1^ to 310 °C, held for 10.25 min. The helium flow rate was 1 mL min^−1^. The mass range of the mass spectrometer was 30–600 *m*/*z* and mass fragments were identified by their respective spectra through NIST v. 14 mass library searches.

## 5. Conclusions

The combination of analytical techniques allowed for the precise identification of the major components present in *Paisaje Abstracto*. The pMMA identified as the binder is not a common polymer employed in artists’ acrylic media alone; instead, the MMA is commonly copolymerized with other monomers and blended with inorganic additives, pigments and fillers. pMMA spherical micro-structures are not observed in other artist’s acrylic formulations, but, nevertheless, these kinds of spheres are commonly observed in dental acrylic cement. Thus, it was possible to propose a hypothesis of the unique painting technique followed by Rafael Coronel to create the mural. The characterization of the polymer suggests a higher pMMA/MMA proportion which may have also affected the mechanical properties and conservation of the mural, causing a rigid painting layer that fractures easily.

## Figures and Tables

**Table 1:** Spectroscopic data (700 MHz, CDCl_3_) of compounds identified in *Paisaje Abstracto* sample. Arrows in green indicate key HMBC correlations and in purple indicate key COSY correlations.

Compound/Structure/Assignment/Correlation	Label	δ_H_/ppm (Multiplicity ^1^, J/Hz)	δ_C_/ppm	HMBC (H→C)
poly(methyl methacrylate) 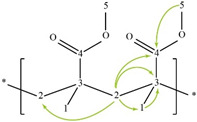	1	0.85 (bs)	16.66	C-2, 3, 4
	1	1.01 (bs)	18.82	C-2, 3, 4
	1	1.21 (bs)	21.24	C-2, 3, 4
	2	1.81 (bs)	51.24–54.48	C-1, 2, 3, 4
	3	---	44.74	-
	4	---	176.26–177.96 178.44	-
	5	3.60 (bs)	51.93	C-4
Methyl methacrylate 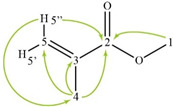	1	3.75 (s)	51.93	C-2
	2	---	167.86	-
	3	---	136.38	-
	4	1.94 (s)	18.49	C-2, 3, 5
	5′	5.56 (s)	125.61	-
	5″	6.10 (s)	125.61	C-2, 4
Benzoyl peroxide 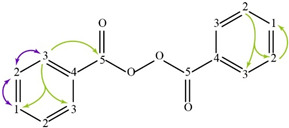	1	7.60 (t, 7.49)	133.69	C-3
	2	7.47 (t, 7.81)	128.63	C-2, 3
	3	8.08 (d, 6.78)	130.28	C-1, 3, 5
	4	---	125.60	-
	5	---	167.79	-
Dibutyl phthalate 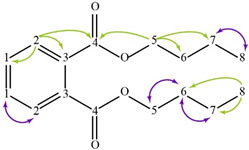	1	7.53 (dd, 5.8, 3.4)	131.03	C-2, 3
	2	7.71 (dd, 5.8, 3.4)	128.92	C-1, 3, 4
	3	---	132.63	-
	4	---	167.91	-
	5	4.31 (t, 6.7)	65.72	C-4, 6, 7
	6	1.72 (q, 6.9)	30.74	C-5, 7, 8
	7	1.44 (h, 7.4)	19.28	C-5, 6, 8
	8	0.96 (t, 7.4)	13.87	C-6, 7
Linear chain alcohol 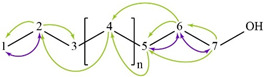	1	0.88 (t, 7.0)	14.2	C-2, 3
	2	1.29 (bs)	22.80	C-1, 3
	3	1.25 (bs)	32.10	C-4
	4	1.25 (bs)	29.40	C-3
	5	1.34 (bs)	25.80	C-4
	6	1.56 (bs)	32.89	C-4, 5, 7
	7	3.64 (t, 6.7)	63.27	C-5, 6
PR181 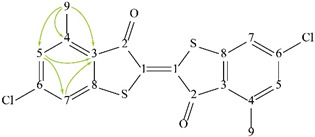	1	---	NA ^2^	-
	2		NA ^2^	
	3	---	125.09	-
	4	---	143.63	-
	5	7.09 (s)	129.05	C-3, 7, 9
	6	---	---	-
	7	7.36	121.98	C-3, 5
	8	---	NA ^2^	-
	9	2.73	18.85	C-3, 4, 5

^1^ Multiplicity: [s] singlet, [d] doublet, [t] triplet, [q] quintet, [h] hexaplet, [dd] doublet of doublets, [bs] broad signal. ^2^ NA: Not assigned.

**Table 2:** ATR-FTIR absorption bands associated with the analyzed compounds in the *Paisaje Abstacto* sample. The anti-symmetric and symmetric stretching and blending modes are denoted with ν_a_, ν_s_, _a_ and _s_.

Component	Wavenumber (cm^−1^)/[Intensity ^1^]/Assignment	References
poly(methyl methacrylate)	749 [m] ν(C-C) skeletal mode, 842 [m] γ(CH_2_), 967 [sh] γ(α-CH_3_), 988 [m] γ(O-CH_3_), 1063 [m] ν(C-C) skeletal mode, 1144 [vs] and 1190 [vs] ν_a_(C-O-C-), 1240 [s] and 1270 [s] ν(C-O), 1386 [m] δ_s_(C-H) of α-CH_3_, 1434 [s] δ_s_(C-H) of O-CH_3_, 1446 [s] δ_a_(C-H) of α-CH_3_, 1482 [m] δ(CH_2_), 1722 [vs] ν(C=O), 2847 [vw] and 2922 [sh] combination band involving O-CH_3_ and CH_2_, 2948 [m] ν_s_(C-H) of O-CH_3_ with ν_s_(C-H) of α-CH_3_ and ν_a_(C-H), 2994 [m] ν_a_(C-H) of O-CH_3_ and ν_a_(C-H) of α-CH_3_.	[11,12]
Red Pigment PR 181	1654 [vs], 1562 [m], 1434 [s], 1386 [w], 1294 [sh], 1240 [w], 1190 [sh], 1096 [sh], 1048 [sh], 842 [vw], 823 [vw], 781 [vw], 468 [vw].	[13,14]
Cadmium sulfide (CdS)	1114 [sh], 700 [vw], 669 [vw], 607 [vw], 572 [vw]	[15,16,17,18]

^1^ Intensity: [vs] very strong, [s] strong, [m] medium, [w] weak, [vw] very weak, [sh] shoulder.

## Data Availability

Not applicable.

## Supplementary Materials

The following supporting information can be downloaded at: https://www.mdpi.com/article/10.3390/molecules28020564/s1, Figure S1: (**a**) SEM micrograph (BSE, 17.0 kV, 1500×). (**b**) Elemental mappings (EDS, 17.0 kV, 1500×) of predominant elements: (**c**) C, (**d**) O, (**e**) Cl, (**f**) Cd, (**g**) Ti, (**h**) S, (**i**) Al and (**j**) Si; Figure S2: ^1^H-NMR spectra (700 MHz, CDCl_3_) of isolated layers: (a) yellow and (b) red. Structures and colored signals of the binder (blue), MMA monomer (violet), catalyst (green), plasticizer (orange), additive (cyan) and red pigment PR181, are indicated; Figure S3: ^13^C-NMR spectra (175 MHz, CDCl_3_) of isolated yellow layer; Figure S4: HSQC (blue-orange) and HMBC (green) spectra (700 MHz, CDCl_3_) of isolated yellow layer. The structure of the acrylic binder pMMA with the key correlation and the assignment of the signals in the spectra are shown; Figure S5: HSQC (blue-orange) and HMBC (green) spectra (700 MHz, CDCl_3_) of isolated yellow layer. The structure of methyl methacrylate monomer with the key correlation and the assignment of the signals in the spectra are shown; Figure S6: COSY spectrum (700 MHz, CDCl_3_) of isolated yellow layer. The structure of benzoyl peroxide (BPO) with the key correlation and the assignment of the signals in the spectrum are shown; Figure S7: HSQC (blue-orange) and HMBC (green) spectra (700 MHz, CDCl_3_) of isolated yellow layer. The structure of benzoyl peroxide (BPO) with the key correlation and the assignment of the signals in the spectra are shown; Figure S8: HSQC (blue-orange) and HMBC (green) spectra (700 MHz, CDCl_3_) of isolated yellow layer. The structure of dibutyl phthalate (DBP) with the key correlation and the assignment of the signals in the spectra are shown; Figure S9: COSY spectrum (700 MHz, CDCl_3_) of yellow layer. The structure of dibutyl phthalate (DBP) with the key correlation and the assignment of the signals in the spectra are shown; Figure S10: COSY spectrum (700 MHz, CDCl_3_) of isolated yellow layer. The structure of 1-octadecanol with the key correlation and the assignment of the signals in the spectra are shown; Figure S11: HSQC (blue-orange) and HMBC (green) spectra (700 MHz, CDCl_3_) of isolated yellow layer. The structure of 1-octadecanol with the key correlation and the assignment of the signals in the spectra are shown; Figure S12: HSQC (blue) and HMBC (green) spectra (700 MHz, CDCl_3_) of isolated red layer. The structure of red pigment PR181 with the key correlation and the assignment of the signals in the spectra are shown; Figure S13: ATR-FTIR spectra obtained from the red layer. The bands associated with the red pigment PR181 are indicated; Figure S14: Chromatogram of isolated red layer. The peaks with the identified compounds are presented at the respective retention times; Figure S15: Mass spectra of the identified compounds in the isolated red layer. (**a**) Benzoyl peroxide, (**b**) dibutyl phthalate, (**c**) 1-octadecanol and (**d**) red pigment PR181.

## Sample Availability

Samples from the mural prepared as cross sections are available from the LANCIC IIE UNAM sample archive for research purposes.
