# Supplementary material for: Microanalytical Characterization of an Innovative Modern Mural Painting Technique by SEM-EDS, NMR and Micro-ATR-FTIR among Others
Source: Molecules. 2023 Jan 5;28(2):564. doi: 10.3390/molecules28020564 (PMC9861809; doi:10.3390/molecules28020564)
Supplement: Supplementary file 1 [file molecules-28-00564-s001.zip › molecules-2098463-supplementary.pdf]

Microanalytical characterization of an innovative modern mural painting technique by SEM-EDS, NMR and micro-ATR-FTIR among others

Pablo Aguilar-Rodríguez, [1] Sandra Zetina, [2] Adrián Mejía-González, [1] Nuria Esturau-Escofet. [1*]

[1] Instituto de Química, Universidad Nacional Autónoma de México, Mexico City 04510, Mexico

[2] Instituto de Investigaciones Estéticas, Universidad Nacional Autónoma de México, Mexico City 04510, Mexico

* Correspondence: nesturau@iquimica.unam.mx

Figure S1. a) SEM micrograph (BSE, 17.0 kV, 1500x). b) Elemental mappings (EDS, 17.0 kV, 1500x) of predominant elements: c) C, d) O, e) Cl, f) Cd, g) Ti, h) S, i) Al and j) Si. 3
Figure S2. ^{1}H-NMR spectra (700 MHz, $CDCl_3$) of isolated layers: a) yellow and b) red. Structures and colored signals of the binder (blue), MMA monomer (violet), catalyst (green), plasticizer (orange), additive (cyan) and red pigment PR181, are indicated. 5
Figure S3. ^{13}C-NMR spectra (175 MHz, $CDCl_3$) of isolated yellow layer. 5
Figure S4. HSQC (blue-orange) and HMBC (green) spectra (700 MHz, $CDCl_3$) of isolated yellow layer. The structure of the acrylic binder pMMA with the key correlation and the assignment of the signals in the spectra are shown. 6
Figure S5. HSQC (blue-orange) and HMBC (green) spectra (700 MHz, $CDCl_3$) of isolated yellow layer. The structure of methyl methacrylate monomer with the key correlation and the assignment of the signals in the spectra are shown. 7
Figure S6. COSY spectrum (700 MHz, $CDCl_3$) of isolated yellow layer. The structure of benzoyl peroxide (BPO) with the key correlation and the assignment of the signals in the spectrum are shown. 8
Figure S7. HSQC (blue-orange) and HMBC (green) spectra (700 MHz, $CDCl_3$) of isolated yellow layer. The structure of benzoyl peroxide (BPO) with the key correlation and the assignment of the signals in the spectra are shown. 9
Figure S8. HSQC (blue-orange) and HMBC (green) spectra (700 MHz, $CDCl_3$) of isolated yellow layer. The structure of dibutyl phthalate (DBP) with the key correlation and the assignment of the signals in the spectra are shown. 10
Figure S9. COSY spectrum (700 MHz, $CDCl_3$) of yellow layer. The structure of dibutyl phthalate (DBP) with the key correlation and the assignment of the signals in the spectra are shown. 11
Figure S10. COSY spectrum (700 MHz, $CDCl_3$) of isolated yellow layer. The structure of 1-octadecanol with the key correlation and the assignment of the signals in the spectra are shown. 12
Figure S11. HSQC (blue-orange) and HMBC (green) spectra (700 MHz, $CDCl_3$) of isolated yellow layer. The structure of 1-octadecanol with the key correlation and the assignment of the signals in the spectra are shown. 13
Figure S12. HSQC (blue) and HMBC (green) spectra (700 MHz, $CDCl_3$) of isolated red layer. The structure of red pigment PR181 with the key correlation and the assignment of the signals in the spectra are shown. 14
Figure S13. ATR-FTIR spectra obtained from the red layer. The bands associated with the red pigment PR181 are indicated. 15
Figure S14. Chromatogram of isolated red layer. The peaks with the identified compounds are presented at the respective retention times 15
Figure S15. Mass spectra of the identified compounds in the isolated red layer. a) Benzoyl peroxide, b) dibutyl phthalate, c) 1-octadecanol and d) red pigment PR181. 16

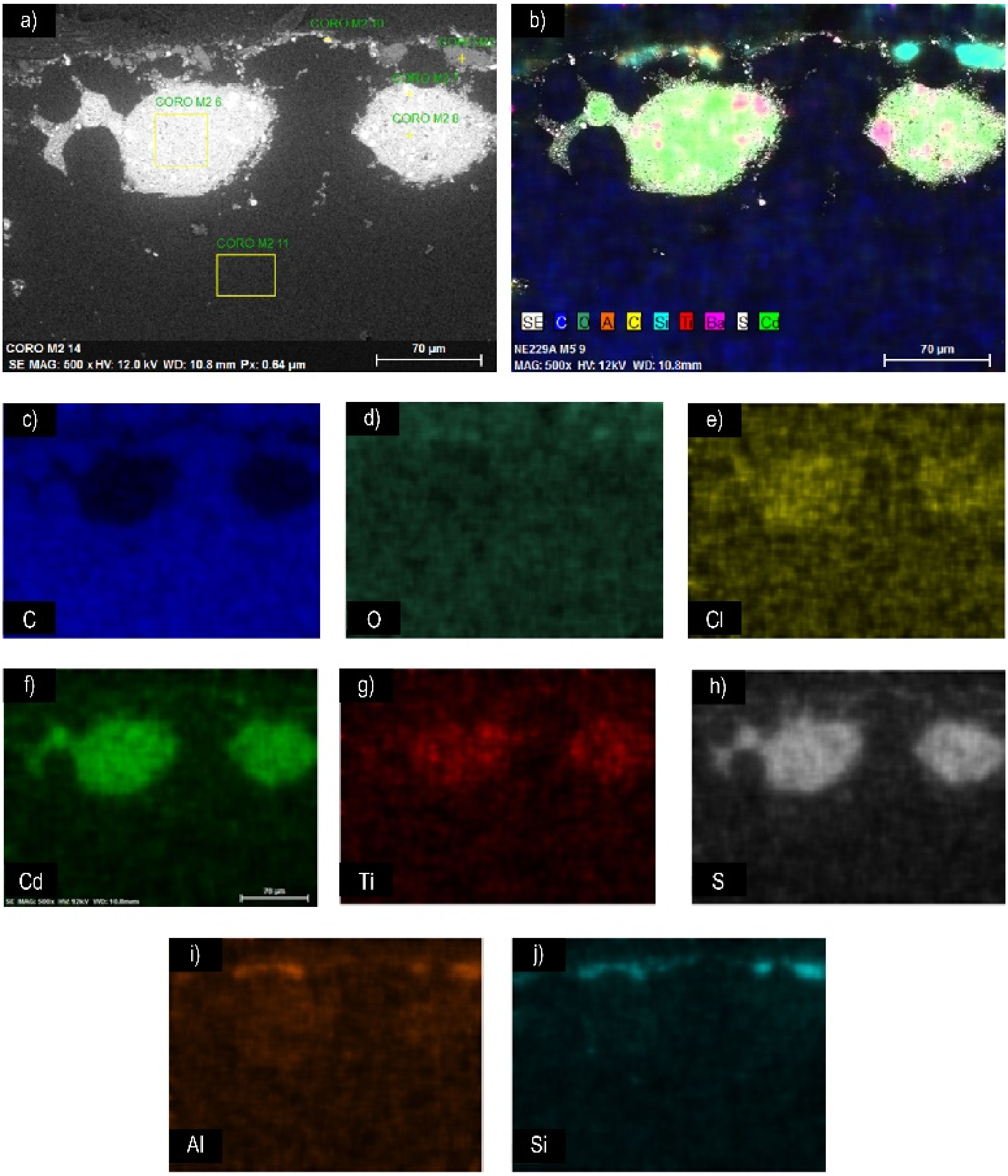

Figure S1. a) SEM micrograph (BSE, 17.0 kV, 1500x). b) Elemental mappings (EDS, 17.0 kV, 1500x) of predominant elements: c) C, d) O, e) Cl, f) Cd, g) Ti, h) S, i) Al and j) Si.

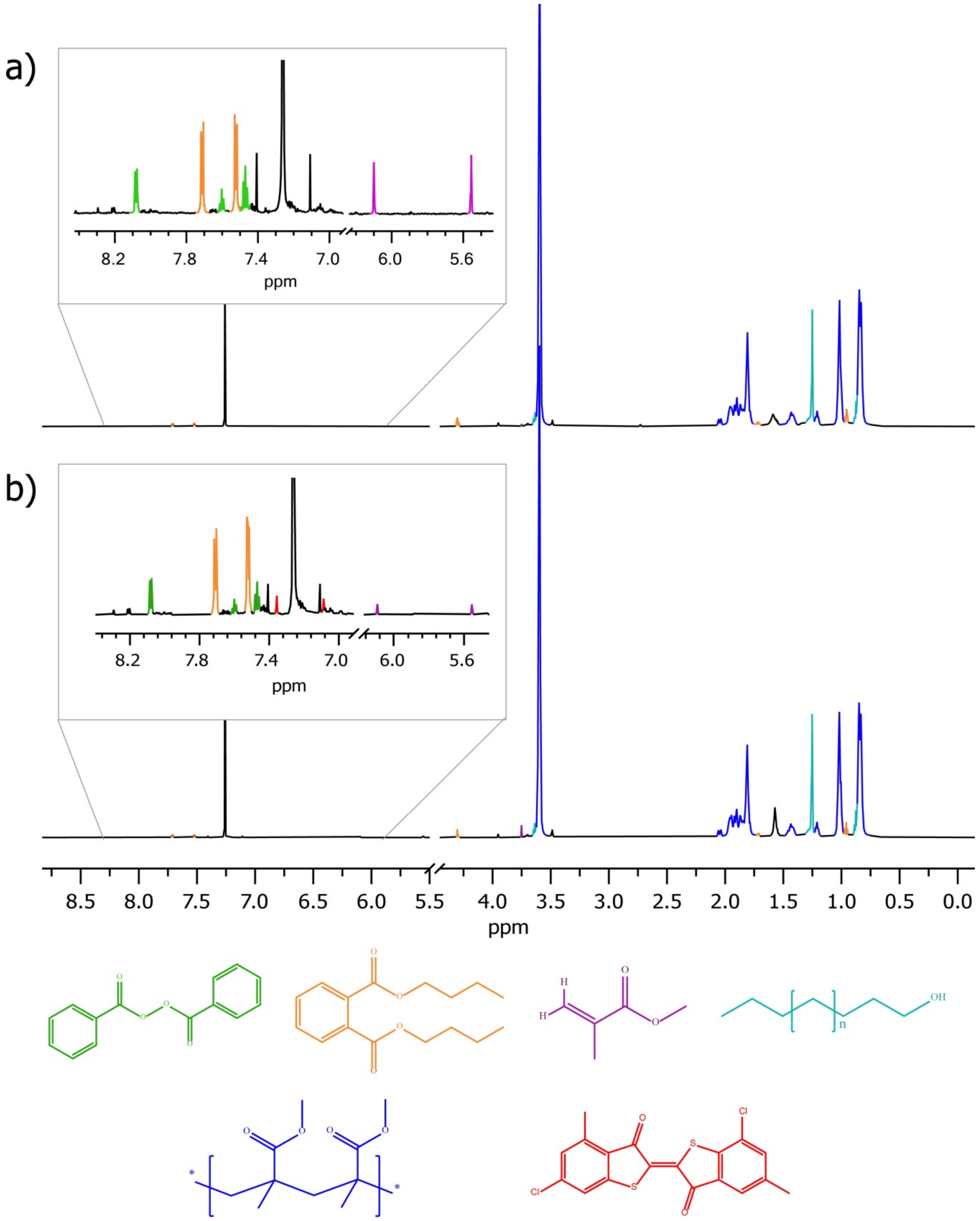
a)
b)
8.2
7.8
7.4
7.0
6.0
5.6
ppm
8.5
8.0
7.5
7.0
6.5
6.0
5.5
4.0
3.5
3.0
2.5
2.0
1.5
1.0
0.5
0.0
ppm

Figure S2. ^{1}H-NMR spectra (700 MHz, $CDCl_3$) of isolated layers: a) yellow and b) red. Structures and colored signals of the binder (blue), MMA monomer (violet), catalyst (green), plasticizer (orange), additive (cyan) and red pigment PR181, are indicated.

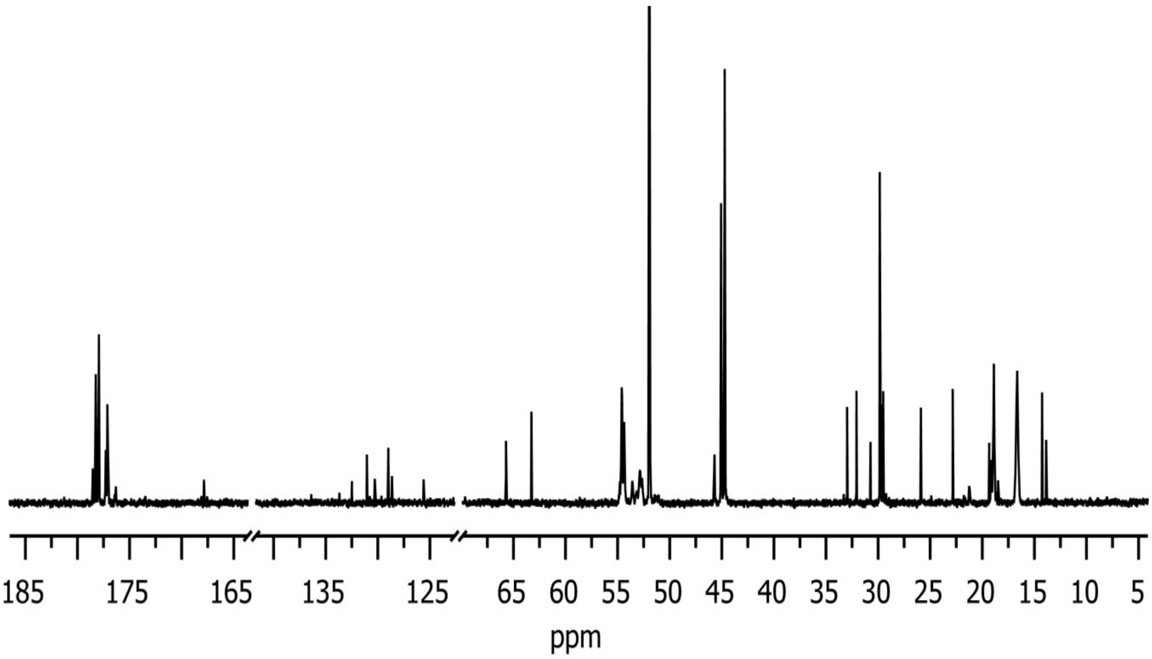

Figure S3. ^{13}C-NMR spectra (175 MHz, $CDCl_3$) of isolated yellow layer.

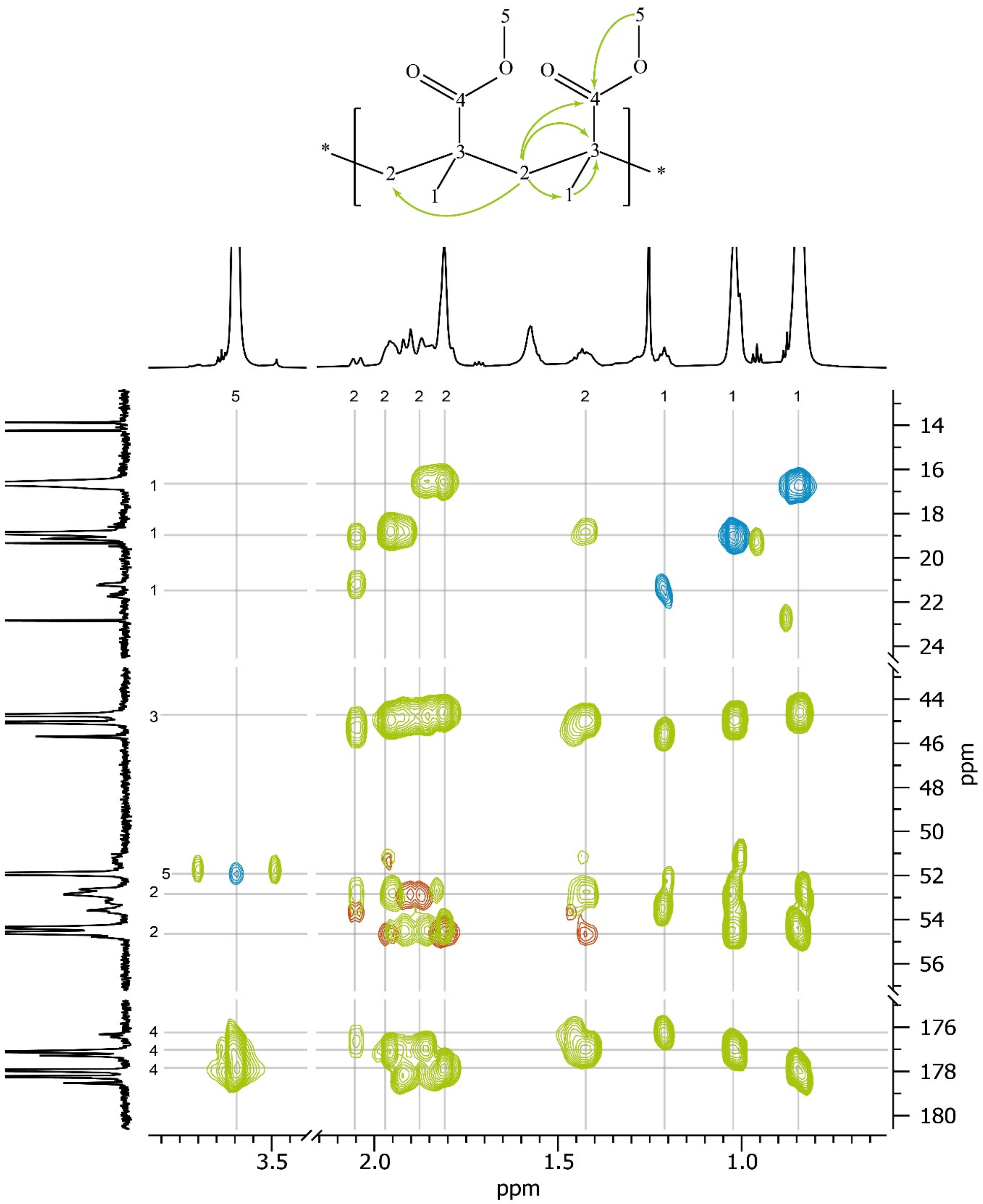

Figure S4. HSQC (blue-orange) and HMBC (green) spectra (700 MHz, $CDCl_3$) of isolated yellow layer. The structure of the acrylic binder pMMA with the key correlation and the assignment of the signals in the spectra are shown.

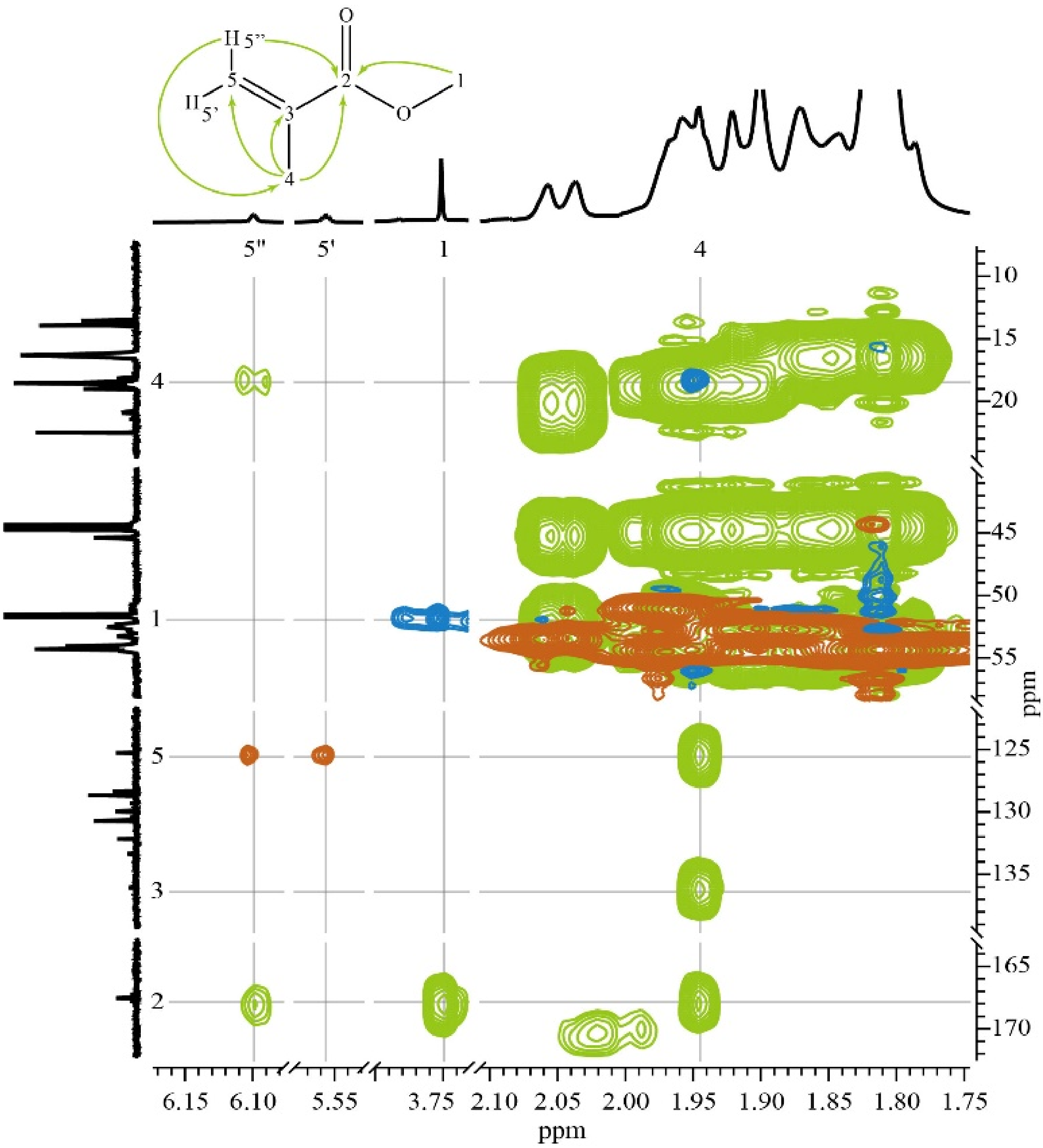

Figure S5. HSQC (blue-orange) and HMBC (green) spectra (700 MHz, $CDCl_3$) of isolated yellow layer. The structure of methyl methacrylate monomer with the key correlation and the assignment of the signals in the spectra are shown.

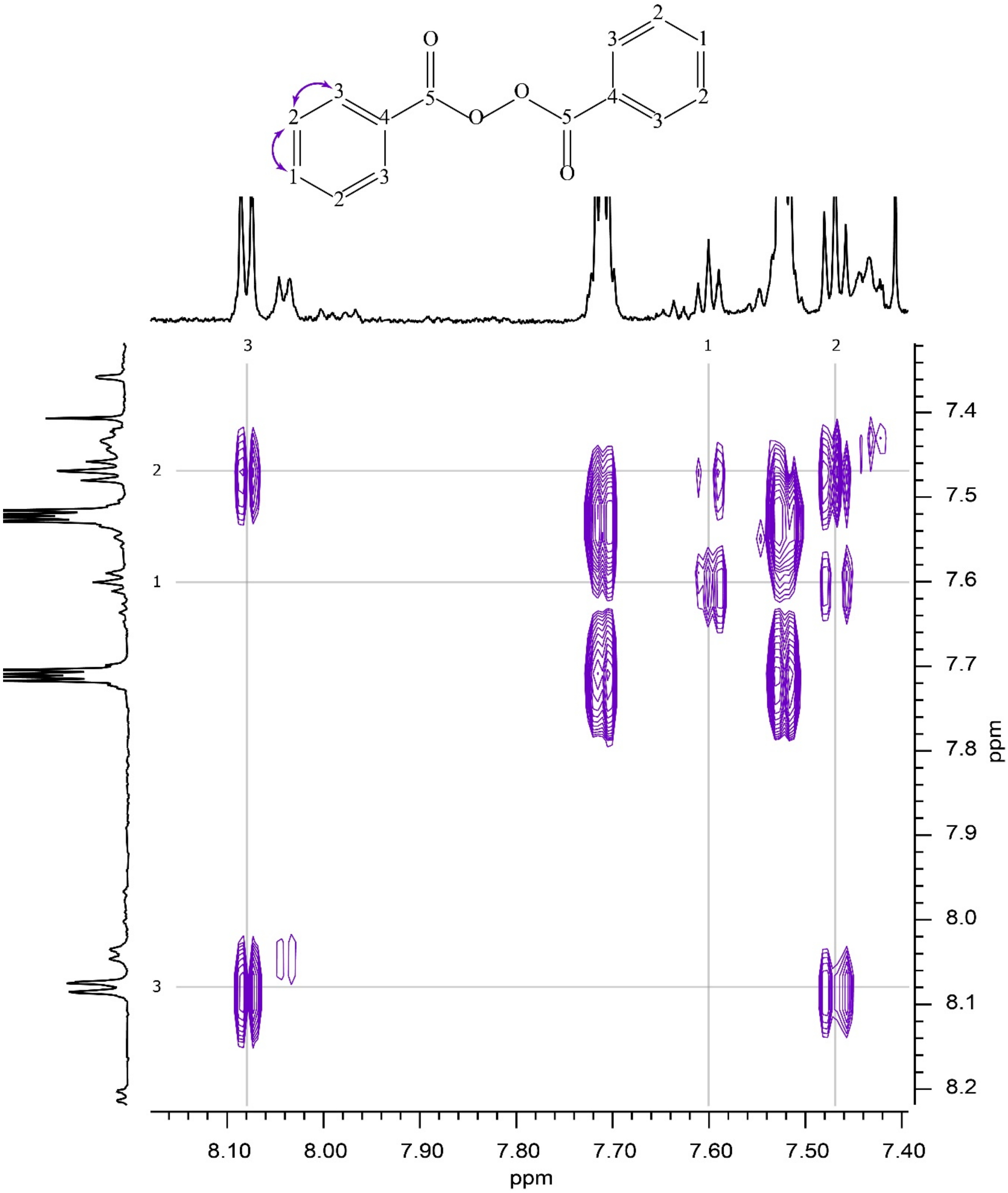

Figure S6. COSY spectrum (700 MHz, $CDCl_3$) of isolated yellow layer. The structure of benzoyl peroxide (BPO) with the key correlation and the assignment of the signals in the spectrum are shown.

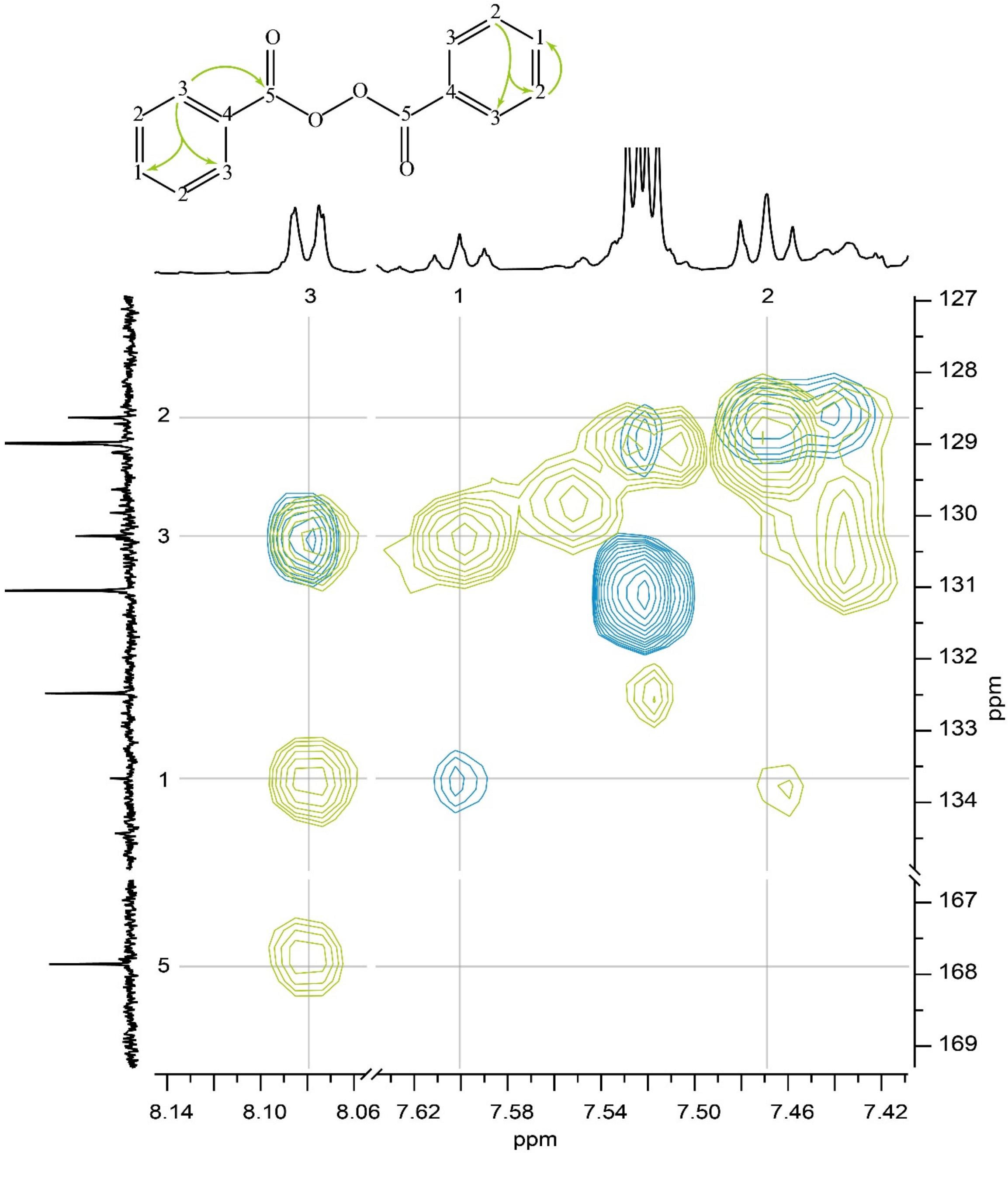

Figure S7. HSQC (blue-orange) and HMBC (green) spectra (700 MHz, $CDCl_3$) of isolated yellow layer. The structure of benzoyl peroxide (BPO) with the key correlation and the assignment of the signals in the spectra are shown.

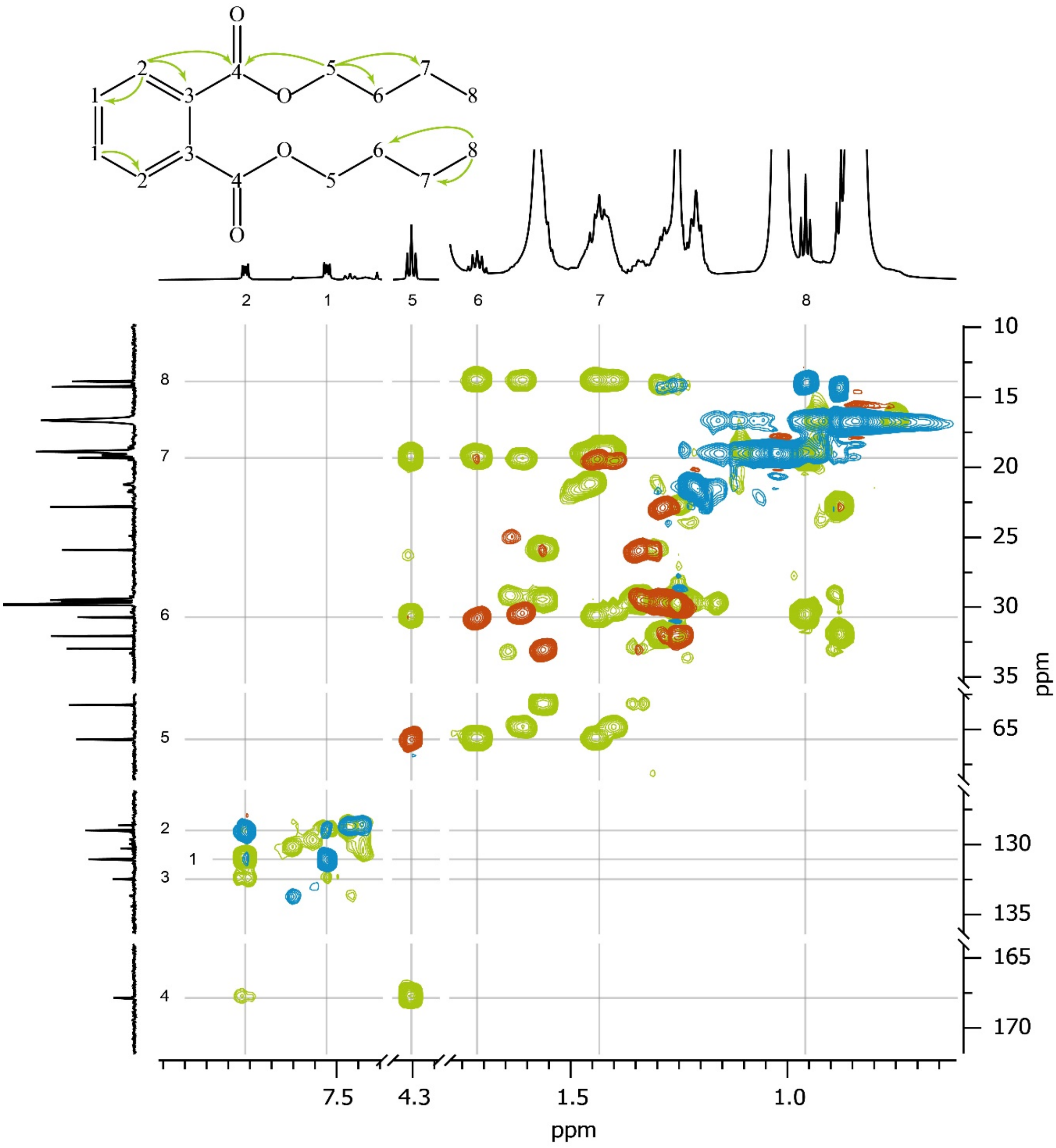

Figure S8. HSQC (blue-orange) and HMBC (green) spectra (700 MHz, $CDCl_3$) of isolated yellow layer. The structure of dibutyl phthalate (DBP) with the key correlation and the assignment of the signals in the spectra are shown.

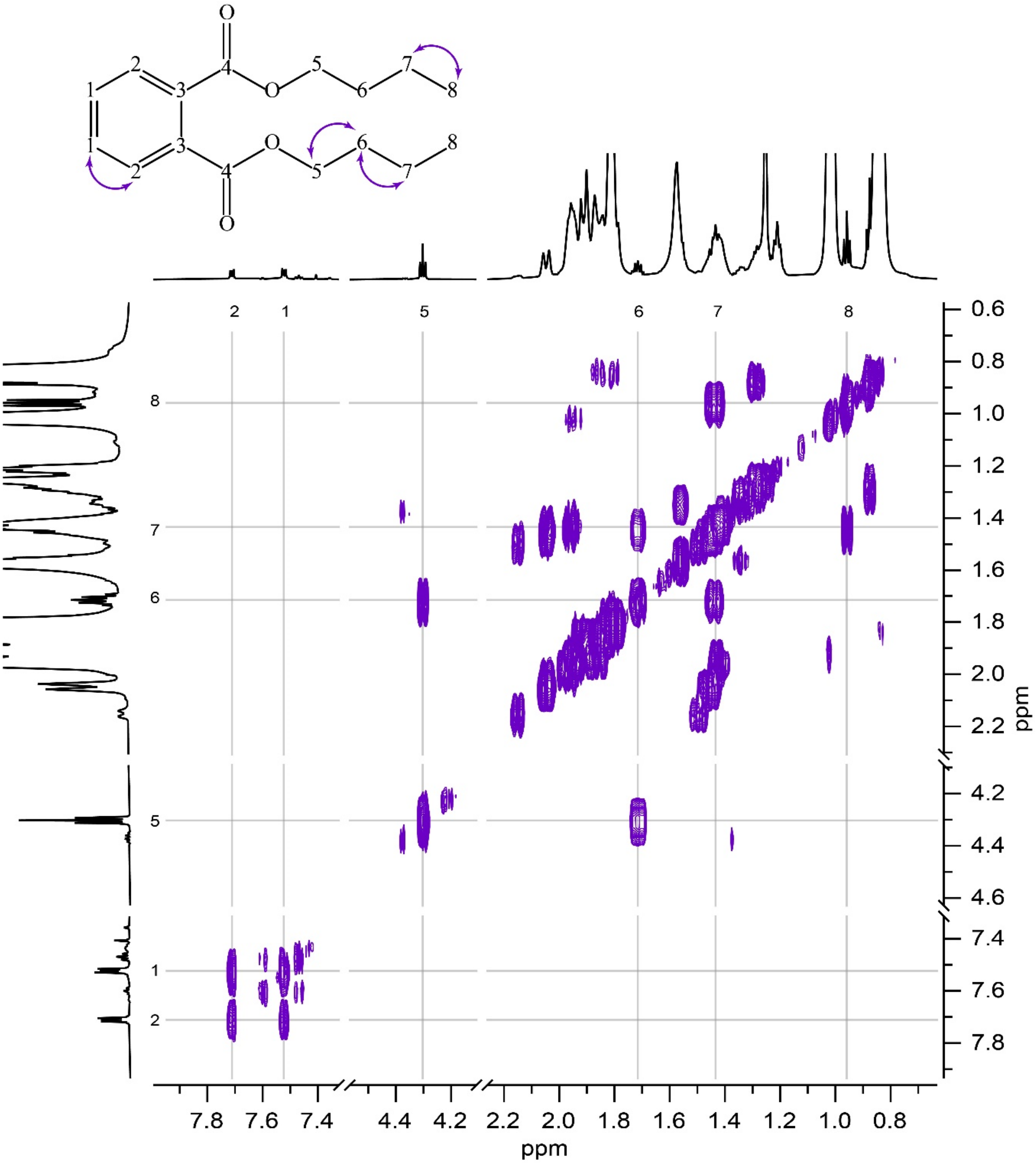

Figure S9. COSY spectrum (700 MHz, $CDCl_3$) of yellow layer. The structure of dibutyl phthalate (DBP) with the key correlation and the assignment of the signals in the spectra are shown.

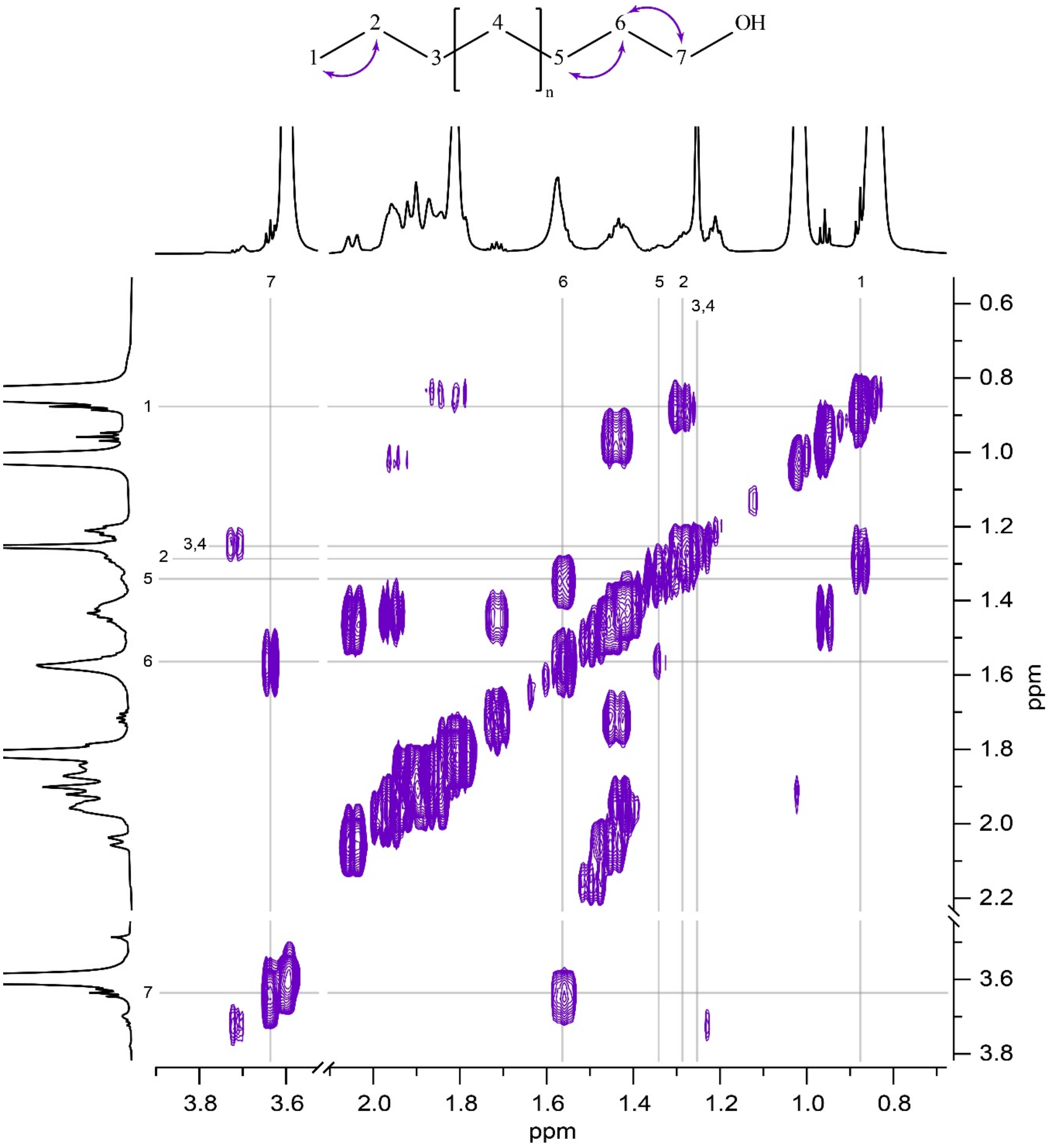

Figure S10. COSY spectrum (700 MHz, $CDCl_3$) of isolated yellow layer. The structure of 1-octadecanol with the key correlation and the assignment of the signals in the spectra are shown.

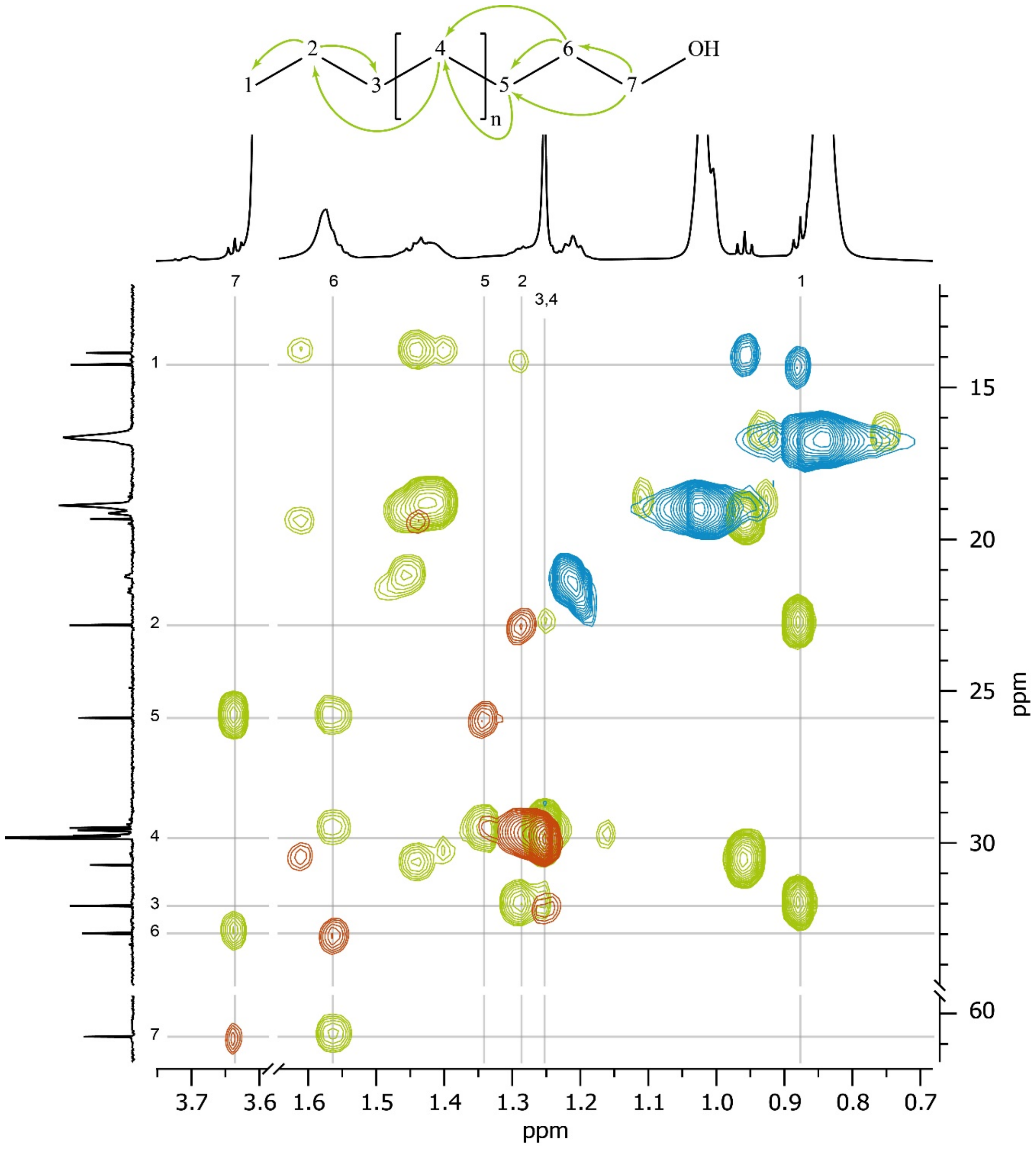

Figure S11. HSQC (blue-orange) and HMBC (green) spectra (700 MHz, $CDCl_3$) of isolated yellow layer. The structure of 1-octadecanol with the key correlation and the assignment of the signals in the spectra are shown.

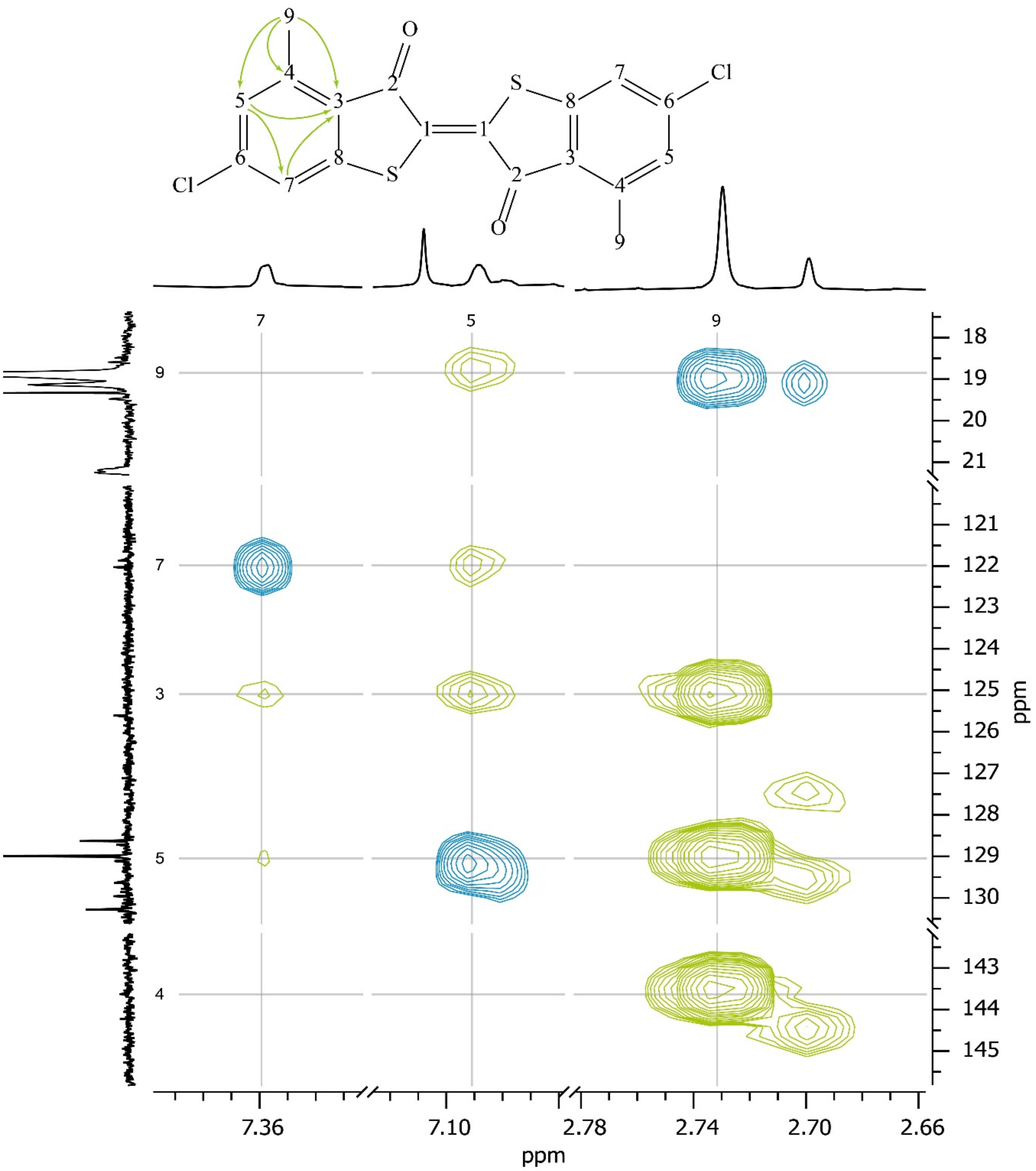

Figure S12. HSQC (blue) and HMBC (green) spectra (700 MHz, $CDCl_3$) of isolated red layer. The structure of red pigment PR181 with the key correlation and the assignment of the signals in the spectra are shown.

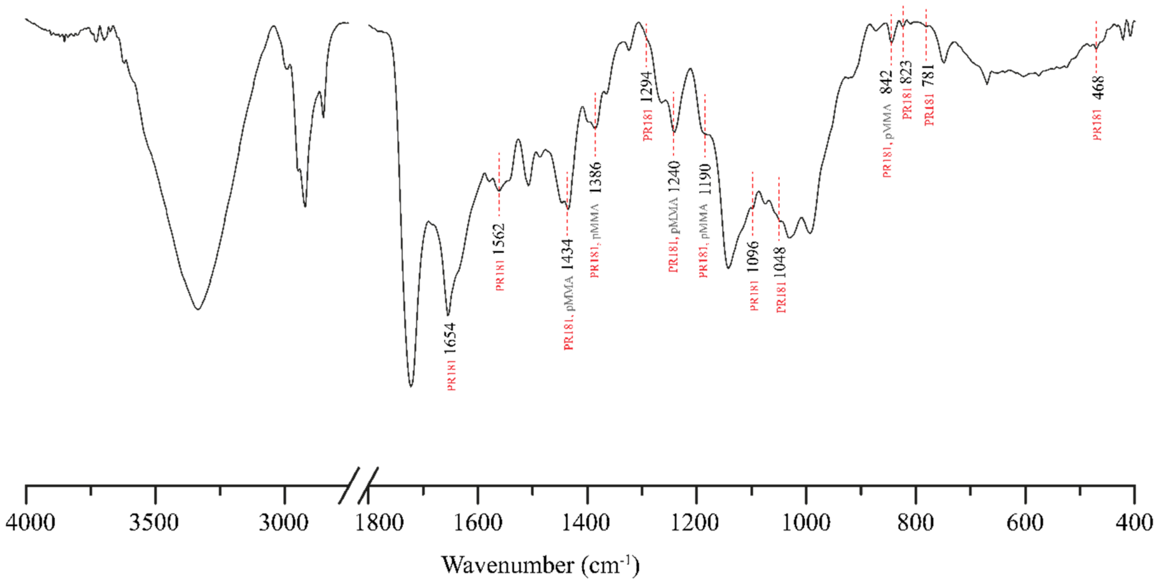

Figure S13. ATR-FTIR spectra obtained from the red layer. The bands associated with the red pigment PR181 are indicated.

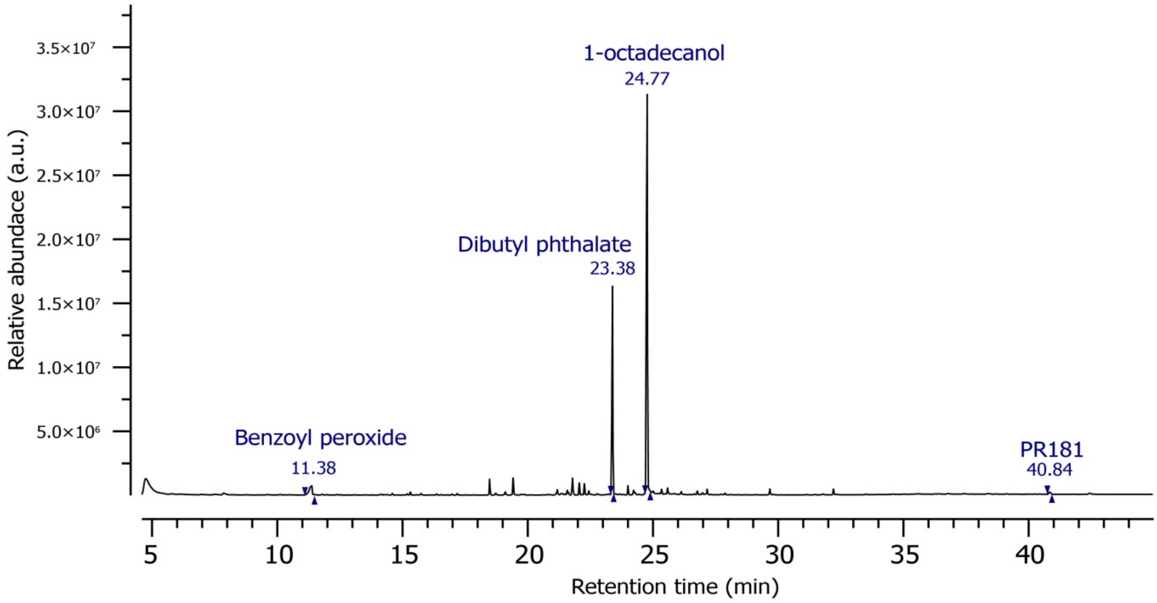

Figure S14. Chromatogram of isolated red layer. The peaks with the identified compounds are presented at the respective retention times.

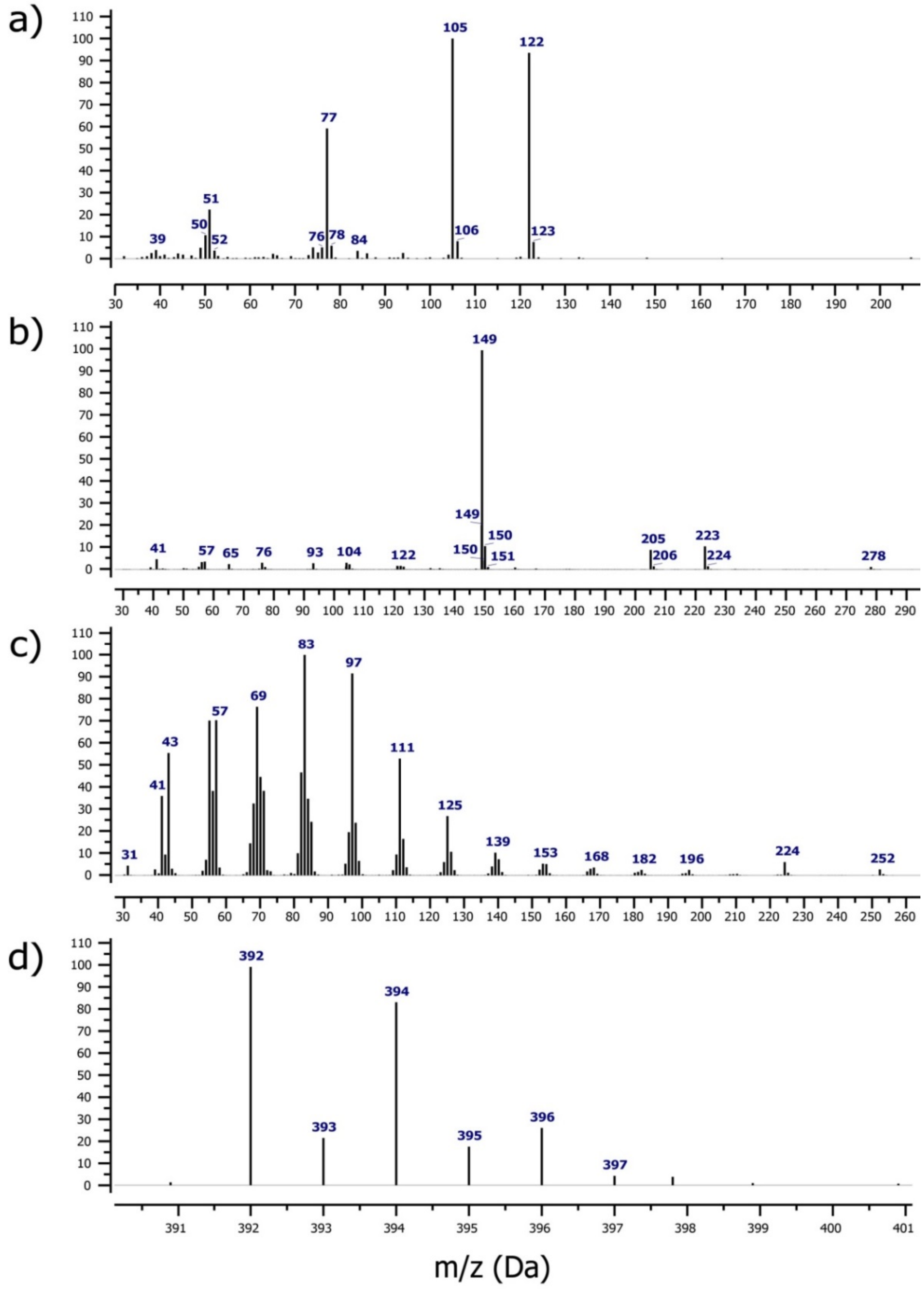

Figure S15. Mass spectra of the identified compounds in the isolated red layer. a) Benzoyl peroxide, b) dibutyl phthalate, c) 1-octadecanol and d) red pigment PR181.
